# An internet-based self-help intervention for older adults after marital bereavement, separation or divorce: study protocol for a randomized controlled trial

**DOI:** 10.1186/s13063-016-1759-5

**Published:** 2017-01-13

**Authors:** Jeannette Brodbeck, Thomas Berger, Hans Joerg Znoj

**Affiliations:** Department of Psychology, University of Bern, Fabrikstrasse 8, 3012 Bern, Switzerland

**Keywords:** Bereavement, Depression, Divorce, Grief, Internet-based self-help, Older adults, Randomized controlled trial, Separation

## Abstract

**Background:**

Marital bereavement and separation or divorce are among the most stressful critical life events in later life. These events require a dissolution of social and emotional ties, adjustments in daily routine and changes in identity and perspectives for the future. After a normative grief or distress reaction, most individuals cope well with the loss. However, some develop a prolonged grief reaction. Internet-based self-help interventions have proved beneficial for a broad range of disorders, including complicated grief. Based on the task model and the dual-process model of coping with bereavement, we developed a guided internet-based self-help intervention for individuals who experienced marital bereavement, separation or divorce at least 6 months prior to enrolment. The intervention consists of 10 text-based self-help sessions and one supportive email a week. The primary purpose of this study is the evaluation of the feasibility and efficacy of the intervention compared with a waiting control group. The secondary purpose is to compare the effects in bereaved and separated participants. Furthermore, we aim to analyze other predictors, moderators and mediators of the outcome, such as age, psychological distress and intensity of use of the intervention.

**Methods:**

The design is a randomized controlled trial with a waiting control condition of 12 weeks and a 24-weeks follow-up. At least 72 widowed or separated participants will be recruited via our study website and internet forums. Primary outcomes are reductions in grief symptoms, depression and psychological distress. Secondary outcome measures are related to loneliness, satisfaction with life, embitterment and the sessions.

**Discussion:**

The trial will provide insights into the acceptance and efficacy of internet-based interventions among adults experiencing grief symptoms, psychological distress and adaptation problems in daily life after spousal bereavement, separation or divorce. Findings will add to existing knowledge by (1) evaluating an internet-based intervention specifically designed for spousal bereavement and its consequences; (2) testing whether this intervention is equally effective for individuals after separation or divorce; and (3) suggesting adaptations to improve the efficacy of the intervention, selective indication and adaptations for different needs.

**Trial registration:**

ClinicalTrials.gov, NCT02900534. Registered on 1 September 2016.

**Electronic supplementary material:**

The online version of this article (doi:10.1186/s13063-016-1759-5) contains supplementary material, which is available to authorized users.

## Background

Marital bereavement and separation or divorce are among the most stressful critical life events in later life. Both events imply a dissolution of social and emotional ties. This deeply affects the attachment system, and requires acceptance of the loss as well as the formation of a new identity and a new perspective for the future. Both events involve the adaptation of daily routines, which can be even more challenging when social, physical and financial resources decline in later life [1].

Grief and psychological distress after bereavement or divorce are normative reactions. For most people, grief intensity weakens to a manageable degree within several weeks or months. After the most intensive period, grief is still present but the loss becomes gradually integrated and no longer hinders the processes of ongoing life. However, some individuals are less able to cope with bereavement or divorce and show severe prolonged grief symptoms or adaptation problems lasting more than 6 months [2–5]. Some individuals even develop a persistent complex bereavement disorder, which is characterized by separation distress, frequent or disabling cognitive, emotional and behavioural symptoms, such as avoidance of reminders of the loved one, difficulties moving on with life and functional impairment [6, 7].

Several theoretical models describe factors that are crucial for an adaptive adjustment to bereavement. The task model identifies four tasks of mourning, namely, accepting the reality of the loss, experiencing the pain of grief, adjusting to an environment without the deceased person, and withdrawing emotional energy and reinvesting it in another relationship [8]. The dual-process model of coping with bereavement posits that a dynamic coping process oscillating between loss-oriented tasks, such as grief work, and restoration-oriented tasks, such as attending to life changes, is essential for adjustment [9]. Coping with loss-oriented tasks involves positive reappraisal versus rumination, revisions of personal goals, positive and negative event interpretation, and expressing emotions toward the deceased. Restoration-oriented coping is focused on attending to life changes, engaging in new activities, distracting from grief, and finding new roles and identities.

These models also provide a theoretical background for interventions ranging from self-help groups and pastoral care to psychotherapy. Cognitive-behavioural interventions for complicated grief are often based on three components: (1) exposure, e.g. the confrontational technique of ‘revisiting’ the deceased person or telling the story of the loss; (2) cognitive reappraisal or restructuring of individual dysfunctional thoughts (e.g., guilt, anger) associated with the loss; and (3) integration and restoration [10, 11]. Internet-based interventions increasingly complement grief counselling or therapy [12–15]. The majority of internet interventions combine the presentation of a web-based self-help programme with minimal but regular therapist contact. In a recent meta-analysis, this internet-based guided self-help approach has proved to be as effective as face-to-face therapy for depressive symptoms, social anxiety disorder and other psychological or somatic disorders [16]. Furthermore, internet-based interventions have advantages over face-to-face therapy. Benefits of internet-based approaches are low threshold accessibility, flexible usage, independent of time and place, usage at a self-determined pace, a high level of autonomy and privacy, and lower costs [17]. These factors may be especially relevant for older adults. However, challenges of internet-based interventions include technological problems and lower computer literacy or unease using computers, which may be more prevalent in old age.

Exposure, cognitive reappraisal, and integration and restoration as treatment components have also been implemented and evaluated in two randomized controlled trials of internet-based self-help interventions for complicated grief after bereavement. One 5 week internet-based intervention consisted of two writing assignments a week of approximately 45 min [12]. After every second assignment, participants received an email from a therapist with personal feedback and further instructions. This intervention addressed individuals who experienced symptoms of intrusion, avoidance, or maladaptive behaviour after the death of a significant other. The average age of the 55 participants was 37 years; all were women; 61% had lost a child and 10% their spouses. Effect sizes (Cohens *d*) for the comparison with the waiting group ranged from 0.96–1.74 for different outcome measures. Follow-up measures at 18 months confirmed the stability of these effects [18].

Another internet-based intervention comprised five structured confrontational writing assignments for individuals who experienced the death of a first-degree relative and who were significantly distressed [13]. The average age of the 757 participants was 43 years; 94% were women; 43% lost a child and 30% their spouses. Effect sizes ranged from 0.19 for emotional loneliness to 0.30 for positive mood for short-term follow-up and 0.25 and 0.23 for long-term follow-ups. These effects were mediated by lower rumination. However, grief and depressive symptoms did not improve. Risk and baseline distress were not confirmed as moderators.

In contrast with these two studies, Litz and colleagues evaluated an internet-based intervention focusing on self-care, social reengagement and goal-focused activities [14]. No formal exposure or cognitive reappraisal was included. Their randomized controlled trial targeted participants between 3 and 6 months after loss and aimed at exploring whether their intervention could prevent prolonged grief disorder. The intervention consisted of 18 sessions covering about 6 weeks, an initial phone call, and periodic brief emails from a therapist. The average age of the 84 participants was 55 years; 68% were women; 78% lost their spouses. The intervention resulted in Cohens *d* of 1.10 for the reduction in prolonged grief, 0.71 for depression and 0.51 for anxiety.

Finally, a recent study compared an internet-based exposure and behavioural activation treatment [15]. The therapist-guided interventions consisted of six homework assignments over 6–8 weeks and a short feedback after each assignment. The 47 participants were randomly allocated to the two active treatment conditions and a waiting control group. The mean age was 46 years; 92% were women; 40% reported the death of a partner and 60% reported other losses. Both interventions reduced complicated grief, post-traumatic stress, and grief rumination, but only exposure had an effect on depression and brooding levels relative to the control group. Effect sizes ranged between *d* = 0.07 and *d* = 1.2. The effects of both interventions were maintained at the 3-month follow-up assessment. To the best of our knowledge, no internet-based self-help intervention has been evaluated for divorced individuals.

### Objectives

Based on the task model of mourning, and the dual-process model of coping with bereavement, we developed a guided internet-based self-help intervention called LIVIA. This intervention addresses individuals who experienced marital bereavement or divorce at least 6 months prior to enrolling in the study and are seeking help for coping with prolonged grief symptoms, psychological distress or adaptation problems in daily life.

This study adds to existing knowledge by (1) evaluating an internet-based intervention specifically designed for spousal bereavement and its consequences; (2) testing whether this intervention is equally effective for individuals who suffer from grief and psychological or behavioural adaptation problems after a separation or divorce; and (3) by including loss-oriented tasks, i.e. exposure and cognitive reframing elements, as well as restoration-oriented tasks, i.e. self-care, social reengagement and goal-focused activities. The combination of both components may increase effect sizes compared with previous studies.

The severity of grief symptoms is not a criterion for taking part in the study, but will be analyzed as a moderator variable. We assume that the internet-based self-help intervention leads to beneficial effects across the severity dimension of distress. Individuals who have already developed a prolonged grief disorder, but who are not willing to see a counsellor or therapist, may benefit from the comprehensive internet-delivered intervention. For individuals with less severe distress, a timely intervention may help to prevent the progress from a normal grief or separation reaction to a prolonged grief disorder.

The objectives of the study are:

1. To evaluate the effects of the guided internet-based self-help programme compared with a waiting control condition on:
  Grief symptoms, psychological distress and depression (primary outcomes)
  Loneliness, embitterment, satisfaction with life, and session-related outcomes (secondary outcomes)
2. To analyze moderators for the efficacy of the programme, i.e.:
  Spousal bereavement versus separation or divorce
  Severity of grief symptoms, psychological distress and depression at baseline
  Age, sex and computer literacy
3. To explore mediators for the efficacy of the programme, i.e.:
  Frequency of use of the programme

## Methods

### Study design

**Fig. 1:**
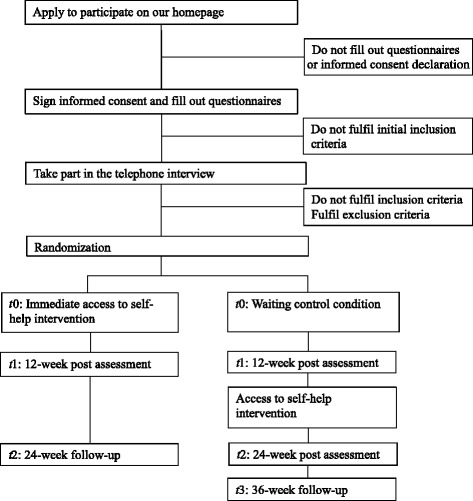
LIVIA flow chart study design

This study is a randomized controlled trial with an internet-based self-help intervention and a waiting control condition. Figure 1 displays the study flowchart. The study population are adults who experienced marital bereavement or a separation or divorce more than 6 months prior to enrolment in the study. The self-help intervention is embedded in a larger Swiss population-based longitudinal study on relationships in later life, the LIVES study, IP 212 [19]. The study coordinator uses two separate lists for widowed and separated individuals, to allocate participants to one of the conditions based on computer generated random numbers using Random.org [20]. Participants in the waiting control group receive access to the intervention 12 weeks after the baseline interview. (The SPIRIT checklist is included as Additional file 1.)

### Sample size

We specified the sample size needed for the different analyses conducting a power analysis based on a probability level of 0.05 and a power of 0.80 with G*Power [21] and a power analysis calculator for structural equation models [22]. To test the efficacy of the intervention compared with the control condition, we expected a large effect of *d* = 0.80. Power analyses indicated a necessary sample size of 42 individuals for this most basic analysis. For the analysis of several predictor variables, as well as moderation and mediation effects using structural equation modelling, we assumed moderate effects of *r* = 0.30. Power analyses resulted in a sample size of 138 participants. Anticipating a drop-out rate of 40% at the 6 month follow-up, we aim at recruiting between 72 and 220 participants at baseline.

### Recruitment

Recruitment is based on the LIVES longitudinal study and internet-self-help forums. All participants who reported difficulties with adjustment after marital bereavement or separation or divorce in the LIVES study receive an information letter about the self-help programme, with a link to the study website, from the LIVES study coordinator. Additionally, information about the intervention with a link to the study website is posted on several internet-based self-help forums.

### Eligibility criteria

All interested adults are required to complete baseline screening questionnaires and a telephone interview for assessing eligibility prior to randomization.

Inclusion criteria are:

1. Experience of marital bereavement or a separation or divorce more than 6 months prior to enrolment in the study
2. Seeking help for coping with prolonged grief symptoms, psychological distress or the psychosocial adaptation to a life without the partner
3. Having access to an internet connection
4. Mastery of the German language
5. Provision of informed consent

Exclusion criteria are:

1. Severe psychological or somatic disorders which need immediate treatment and acute suicidality (Beck Depression Inventory suicide item > 1 or suicidal ideation in the telephone interview).
2. No emergency plan: in the telephone interview, an emergency plan will be developed that specifies a health care professional, to whom participants can turn in an acute crisis. If no such person or health care service can be found, individuals are excluded from the intervention.
3. Concomitant psychotherapy, or prescribed drugs to treat depression or anxiety, if the prescription or dosage has changed in the month prior or during the self-help intervention.
4. Inability to follow the procedures of the study, e.g. due to comprehension problems.

### Description of the intervention

The intervention is a guided 10-week internet-based self-help programme. The 10 text-based sessions are described in detail in Table 1. Participants are encouraged to work through one session a week and to complete the assignments. One session takes between 45 min and 60 min. The first two sessions include general information about interpersonal loss and an assessment of the current personal situation. Sessions 3 to 5 focus on resources and restoration-oriented interventions for fostering positive thoughts and emotions as well as self-care. Sessions 6 and 7 consist of loss-oriented interventions for accepting memories and pain and address unfinished business. Sessions 8 and 9 again include restoration-oriented interventions, focusing on creating a new life without the partner and social relationships. The last session addresses the redefinition of the relationship to the lost person. The sessions for bereaved and separated or divorced participants are identical apart from adaptations in the first session (psychoeducation) and the sixth session (accepting memories and telling the story of the loss).

**Table 1:** Outline of the 10 self-help sessions of the internet-based intervention

1. Psychoeducation	Information about the self-help intervention, grief reactions, reactions to separation, predictors and treatment of complicated grief
2. Assessment of current situation	Information about and assessment of emotions in the context of the interpersonal loss, changes in life since the loss and obstacles for a positive adaptation
3. Fostering positive thoughts and emotions	Information about emotion regulation and cognitive-behavioural strategies to promote positive thoughts and emotions Protocols for practising these strategies in daily life
4. Finding comfort	Suggestions for self-soothing strategies and exercises to promote positive feelings (e.g. diary for positive experiences)
5. Self-care	Checklists for current physical, emotional and practical self-care, formulation of self-care goals and suggestions for implementing self-care behaviour in daily life
6. Accepting memories and pain	Writing tasks to integrate painful memories of the loss into the autobiographical memory and to be able to tell the story of the loss
7. Unfinished business	Identification of unfinished business and regrets, writing tasks to formulate unfinished business and to find ways to put issues at rest
8. Creating a new life without the partner	Identifying changes in daily life since the loss and sources of support and strengths before and after the loss Information about post-traumatic growth Identifying and activating resources in daily life
9. Social relationships	Clarifying current relationships using a sociogram Defining goals related to social relationships, e.g. changing relationships, building up new social contacts, and suggestions for promoting social well-being
10. Redefinition of the relationship to the lost partner	Writing a farewell letter to the lost partner: saying good-bye and telling the lost partner about the future importance of the loss and how the participant will continue life without the lost partner

During the work with the text-based sessions, participants receive email support by psychologists of the Department of Clinical Psychology and Psychotherapy of the University of Bern. These weekly emails acknowledge and motivate participants in their work with the self-help programme and provide a weekly structure and support for technical problems. Participants can contact their supporters anytime with questions via a contact button in the self-help programme. The email-supporters are supervised by a fully trained psychotherapist.

### Measures

**Fig. 2:**
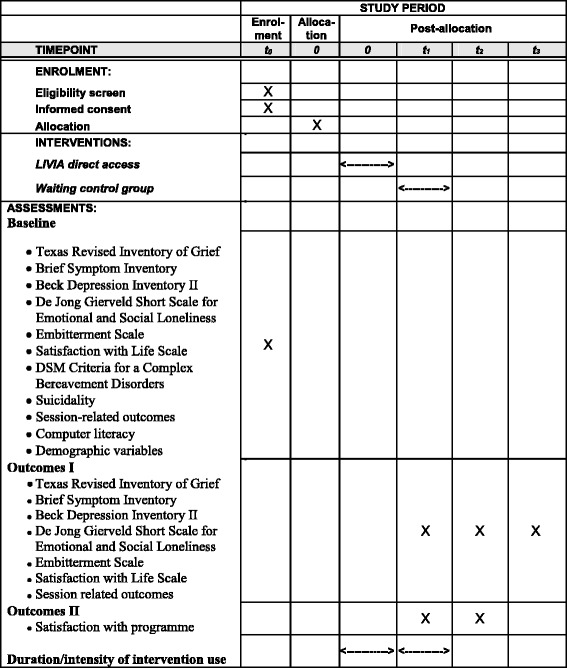
SPIRIT figure: schedule of enrolment, interventions, and assessments

Figure 2 (the SPIRIT figure schedule of enrolment, interventions, and assessments) gives an overview of the measures with the timing of the assessment. All self-report questionnaires are completed online. Baseline measurement is at *t*_0_, post-measurement *t*_1_ is 12 weeks after the start of the programme or waiting condition, post-measurement *t*_2_ is after 24 weeks, and the follow-up measure *t*_3_ will be completed only by the waiting control group 24 weeks after their start of the intervention, i.e. 36 weeks after baseline.

#### Primary outcome measures

Grief symptoms are assessed using the German version of the Texas Revised Inventory of Grief [23]. The Texas Revised Inventory of Grief is a widely used measure to assess the severity of grief symptoms. A recent factor analysis identified three factors for emotional response, thoughts, and non-acceptance regarding a loss [24]. The German version of the Texas Revised Inventory of Grief is a 16-item measure to assess the severity of grief symptoms, from 1 = completely true to 5 = completely false.

Psychopathological distress is assessed using the German version of the Brief Symptom Inventory, a widely used 53-item measure to assess a broad range of somatic and psychopathological symptoms within 7 days prior to completing the questionnaire [25]. Factors include depressed mood, somatic symptoms, information processing deficits, and interpersonal insecurity [26]. Answer categories range from 0 = not at all to 4 = very much.

Depressive symptoms are assessed with the German version of the Beck Depression Inventory II [27]. This measure consists of 21 items on a scale from 0 to 3.

#### Secondary outcome measures

- Loneliness is assessed using the De Jong Gierveld Short Scale for Emotional and Social Loneliness [28]. This is a six-item scale with answer categories from 0 = no to 5 = yes.
- Embitterment is assessed with the short version of the Embitterment Scale, which consists of six items rated on a scale from 0 = I do not agree to 4 = I agree [29].
- Life satisfaction is assessed with the German version of the Satisfaction with Life Scale [30, 31]. It consists of five items with answer categories from 1 = completely disagree to 7 = completely agree.
- Session-related outcomes. We included nine items related to specific sessions of the intervention, e.g. self-rated knowledge about grief symptoms, self-care, self-soothing strategies, satisfaction with social relationships or the current life situation. Items are rated on scale from 1 = very little to 10 = very much.
- Satisfaction with the self-help programme. Six items assess the evaluation of the quality of and the satisfaction with the intervention, and six items assess effects of the intervention related to mechanisms of change, i.e. mastery experiences, clarification experiences or insight, problem actuation, and resource activation. Response categories range from 1 = not at all to 4 = very much.

#### Predictors and moderators

- Computer literacy is assessed using the first seven items of the Computer Literacy Scale for Older Adults [32]. They assess experiences with the computer and the frequency with which participants engage in different computer-related tasks.
- Demographic variables include sex, age, education, overall self-rated health, and details about the marital history and the loss of the partner or the separation or divorce.
- Criteria for a persistent complex bereavement disorder according to the Diagnostic and Statistical Manual of Mental Disorders [7] are assessed in the telephone interview.
- Suicidality is assessed in the telephone interview using the suicidality questions of the Brief Psychiatric Rating Scale [33].
- Adherence and completion data, as well as data on the duration and the intensity of the use of the self-help intervention are collected within the platform.

### Data collection and management

Data are assessed using online-questionnaires programmed in Qualtrics [34]. Data integrity is enforced through a variety of mechanisms, i.e. referential data rules, valid values, range checks, and consistency checks. The option to choose a value from a list of valid codes and a description of what each code means is available where applicable. Checks are applied at the time of data entry into a specific field. In addition, data on the use of the self-help sessions are collected within the platform. All data will be saved in an anonymous way only identified by a code that is not related to the participant’s identity. Servers are protected by high-end firewall systems. Only the researchers directly involved in the study have access to the data.

### Statistical analysis

Analysis will be conducted according to the intention-to-treat paradigm. Firstly, we will analyze the extent of missing data, explore the missing data patterns and determine the type of missing data (missing completely at randomization, missing at randomization, not missing at randomization). If the missing mechanism is missing at randomization, we will use multilevel regression analyses, which allow a different number of measurement points per participants and are thus less sensitive to missing data. We will include time (pre versus post-intervention measures and post-intervention versus follow-up measures), group (immediate access versus control condition), event (bereavement versus separation or divorce) and interaction terms as predictors of the outcome variables. Cohens *d* will be calculated as effect size for all observed outcome variables. To analyze the longitudinal interplay of predictor variables, we will conduct structural equation models. Analysis will be conducted in SPSS and Mplus.

## Discussion

The results of this study will provide insight into the acceptance and efficacy of an internet-based self-help intervention for adults who experience grief symptoms, psychological distress or adjustment problems in daily life after marital bereavement or separation or divorce. The outcomes for bereaved and separated or divorced participants will be compared. The analysis of other moderator variables may further aid future selective indication and adaptations for different needs.

Limitations of this study include the self-selectivity of the sample. It may be possible that older adults who are willing to take part in an internet-based self-help intervention have more cognitive resources and a higher education level.

## Additional file

Additional file 1: SPIRIT 2013 Checklist: recommended items to address in a clinical trial protocol and related documents. (PDF 169 kb)
